# Metabolic Tumor Volume by ^18^F-FDG PET/CT Can Predict the Clinical Outcome of Primary Malignant Spine/Spinal Tumors

**DOI:** 10.1155/2017/8132676

**Published:** 2017-08-09

**Authors:** Yoshihiro Matsumoto, Shingo Baba, Makoto Endo, Nokitaka Setsu, Keiichiro Iida, Jun-Ichi Fukushi, Kenichi Kawaguchi, Seiji Okada, Hirofumi Bekki, Takuro Isoda, Yoshiyuki Kitamura, Hiroshi Honda, Yasuharu Nakashima

**Affiliations:** ^1^Department of Orthopaedic Surgery, Graduate School of Medical Sciences, Kyushu University, 3-1-1 Maidashi, Higashi-ku, Fukuoka 812-8582, Japan; ^2^Department of Clinical Radiology, Graduate School of Medical Sciences, Kyushu University, Japan

## Abstract

**Background and Purpose:**

Primary malignant spine/spinal tumors (PMSTs) are rare and life-threatening diseases. In this study, we demonstrated the advantage of volume-based ^18^F-FDG PET/CT metabolic parameter, metabolic tumor volume (MTV), for assessing the aggressiveness of PMSTs.

**Materials and Methods:**

We retrospectively reviewed 27 patients with PMSTs and calculated SUV_max_, MTV, and total lesion glycolysis (TLG) to compare their accuracy in predicting progression-free survival (PFS) and overall survival (OS) by receiver operating characteristic (ROC) curve analysis. Univariate and multivariate analyses were used to compare the reliability of the metabolic parameters and various clinical factors.

**Results:**

MTV exhibited greater accuracy than SUV_max_ or TLG. The cut-off values for PFS and OS derived from the AUC data were MTV 45 ml and 83 ml and TLG 250 SUV⁎ml and 257 SUV⁎ml, respectively. MTV above cut-off value, but not TLG, was identified as significant prognostic factor for PFS by log-lank test (*p* = 0.04). In addition, MTV was the only significant predictive factors for PFS and OS in the multivariate analysis.

**Conclusions:**

MTV was a more accurate predictor of PFS and OS in PMSTs compared to TLG or SUV_max_ and helped decision-making for guiding rational treatment options.

## 1. Introduction

Primary malignant spine/spinal tumors (PMSTs) are rare tumors and only comprise 4% to 13% of all bone and soft-tissue sarcomas [1]. Management of PMSTs is challenging since those tumors are often inoperable because of the complexity of their surrounding anatomy. Moreover, PMSTs show diverse histological subtypes and degrees of aggressiveness that confuse the treatment of PMSTs. Basically, the clinical behavior of the tumors depends on the aggressiveness of the tumor. Thus, the identification of an aggressive tumor prior to treatment has an essential role in establishment of a rational treatment plan. In different medical fields, various methods that predict a risk of patient and subsequently guide therapy had been reported [2]. However, in the field of spinal oncology, the reports relating to the predictive factors that influence the survival of the PMSTs patients are sparse [3].

Recently, ^18^F-fluoro-deoxy-glucose positron-emission-tomography/computed tomography (^18^F-FDG PET/CT) becomes the gold standard for preoperative assessment of biological activity and malignant capacity of the tumors and the advantages of ^18^F-FDG PET/CT for evaluating histological characteristics, tumor response to treatment, and clinical outcomes in patients with various malignant tumors are reported [4]. In most studies, single pixel values of the maximum standardized uptake value (SUV_max_) have been used as an index of tumor metabolism [5, 6]. However, PMSTs often demonstrate heterogeneous biological activities due to the different histological features of cell proliferation, necrosis, and matrix deposition [7]. On the contrary, SUV_max_ only represents the maximum value of a single voxel in the tumors; thus SUV_max_ may not reflect the true aggressiveness and prognostic properties of the tumors [8].

One of the promising approaches to overcome the shortcomings of SUV_max_ based estimation of aggressiveness of PMSTs is volume-based ^18^F-FDG PET/CT imaging markers such as metabolic tumor volume (MTV) and total lesion glycolysis (TLG) [9]. MTV is defined as the sum of the volume of voxels with SUV surpassing a threshold value in a tumor [9]. TLG is calculated by multiplying MTV and the mean SUV of the MTV [10]. Importantly, recent studies confirm the superiority of MTV and TLG compared to SUV_max_ with regard to prognostic value in head-and-neck cancer, non-small-cell lung cancer, and epithelial ovarian cancer [11, 12]. In contrast, clinical relevance of MTV and TLG in patients with bone and soft-tissue sarcoma remains obscure and controversial [13–15]. To the best of our knowledge, this is the first report that has demonstrated the advantage of metabolic parameters for assessing the aggressiveness of PMSTs.

## 2. Materials and Methods

### 2.1. Patients

We retrospectively reviewed 27 patients with primary malignant spine (19 cases) and spinal (8 cases) tumors. The inclusion criteria were as follows: (1) newly diagnosed and histologically proven PMSTs and (2) having undergone 18F-FDG PET/CT before the initiation of treatment. Patients with previous history of another malignancy, less than 3 months' follow-up, and insufficient clinical data were excluded.

Patient characteristics are summarized in Table 1. The study patients comprised 13 men and 14 women. The median patient age was 53.9 years (range, 12–82 years). Tumor locations included the cervical vertebra (*n* = 7), thoracic vertebra (*n* = 10), lumbar vertebra (*n* = 6), and sacral vertebra (*n* = 4). Maximal lesion diameters ranged from 3.5 to 12 cm; the mean maximal diameter was 6.0 ± 2.8 cm. The maximal diameters of 14 cases were greater than 5 cm. Histological examination showed the following: 5 cases of malignant peripheral nerve sheath tumor, 4 cases of undifferentiated pleomorphic sarcoma, 3 cases of osteosarcoma and chondrosarcoma, 2 cases of chordoma, giant cell tumors of bone, and leiomyosarcoma, and 1 case each of malignant solitary fibrous tumor, malignant myoepithelioma, plasmacytoma, malignant lymphoma, histiocytic sarcoma, and hemangiopericytoma. Sixteen of the 27 cases were managed with surgery (59%). Among the surgically treated cases, tumor resection with wide margin was carried out in 5 patients, while the remaining 11 patients underwent intralesional resection (81%). Various regimens of chemotherapy were followed by 15 patients. Sixteen patients received radiotherapy: 6 received conventional radiotherapy, and 10 received carbon-ion radiotherapy with curative intent. In this study, opt-out method was applied to obtain the consent of the patients and this clinical study was approved by the institutional review board at Kyushu university hospital (26–224).

### 2.2. ^18^F-FDG PET/CT Acquisition and Volumetric Analysis

^18^F-FDG PET/CT acquisition was performed for all patients. In each patient, 4 MBq/kg of ^18^F-FDG was intravenously administered after fasting for at least 4 h. Scans were conducted from the middle of the thigh to the top of the skull 60 min after the ^18^F-FDG administration. Scan range was extended to the extremities as needed according to the location of the primary tumor. ^18^F-FDG PET/CT images were obtained using an integrated PET/CT scanner, the Discovery STE (GE Medical Systems, Milwaukee, WI) or Biograph mCT (Siemens Healthcare). All emission scans were performed in the three-dimensional mode, and the acquisition time per bed position was 3 min for the Discovery STE and 2 min for the Biograph mCT. We reconstructed the PET images using the ordered-subset expectation-maximization method (VUE Point Plus) with two full iterations of 28 subsets for the Discovery STE and iterative True-X algorithm and time of flight (TOF) (Ultra HD-PET) with two full iterations of 21 subsets. The CT scan was reconstructed by filtered backprojection into 512 × 512 pixels' images with a slice thickness of 5 mm to match the PET scan. The PET/CT fusion images were generated using GENIE–Xeleris software on a dedicated work station, Xeleris (GE Medical Systems, Milwaukee, WI).

^18^F-FDG accumulation higher than the background was defined as ^18^F-FDG-positive. The maximum standardized uptake value (SUV_max_) and MTV and TLG in ^18^F-FDG PET images were measured using dedicated software (Multi-Modality Tumor Tracking software; IntelliSpace Portal 6 workstation, Philips Medical Systems, Milpitas, CA). A spherical volume-of-interest (VOI), corresponding to the tumor, was drawn and SUV_max_ for the VOI was automatically calculated. The highest voxel value in the tumor on ^18^F-FDG PET/CT was determined as SUV_max_. Using a SUV of 2.5 as the threshold, the volume of tumor with SUV ≥ 2.5 was determined as MTV (ml), and SUV_mean_ was defined as mean SUV in the delineated tumor volume. The product of the MTV multiplied by SUV_mean_ was defined as TLG (SUV*∗*ml).

### 2.3. Clinical Endpoints

Progression-free survival (PFS) and overall survival (OS) were used as the clinical endpoint to evaluate the prognostic value of the metabolic parameters. PFS was defined as the date of initial treatment to the date of histological or radiological evidence of local recurrence and/or distant metastasis. OS was defined as the time from initial diagnosis to death. For patients without progression or death, the last follow-up time was used as the endpoint.

### 2.4. Statistical Analysis

Receiver operating characteristics (ROC) curve analysis was applied to identify the best discriminating cut-off values for SUV_max_, MTV, and TLG. Appropriate cut-off was defined as the point on the curve nearest to the upper left corner of the ROC graph. The area under the curve (AUC) was used to evaluate the accuracy of the metabolic parameters as a prognostic factor. Kaplan-Meier survival analysis and the log-rank test were used to evaluate the degree of equality of predictive values across variables regarding PFS and OS. A Cox proportional hazards regression model was applied to determine the effect of potential factors that were found significant on univariate and multivariate analysis. Statistical significance was set at *p* < 0.05. JMP version 13 software was used for statistical analysis.

## 3. Results

### 3.1. Clinical Outcome

The median follow-up period was 21.9 months (range 3–58 months, median 18 months). Five patients died of disease during follow-up (17%). Disease progression occurred in 9 patients (31%). Distant metastases and local recurrence were identified in 5 and 8 patients, respectively. Four patients experienced both local and distant progression. The probabilities of 2-year PFS and overall survival were 66% and 81%, respectively.

### 3.2. ROC Curve Analysis, AUC, and Cut-Off Values

**Figure 1:**
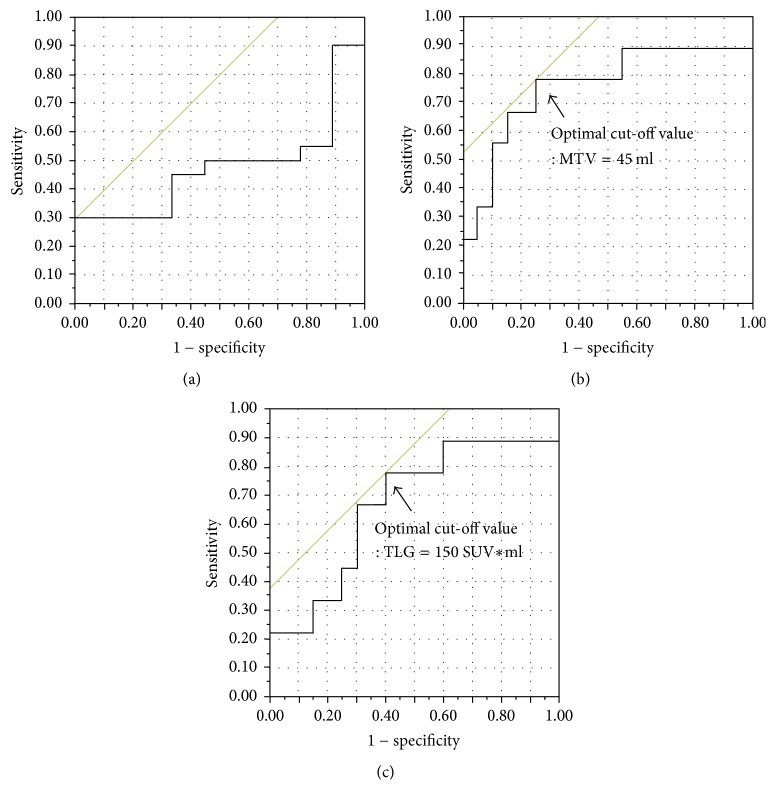
*ROC curve analysis comparing the prognostic accuracy for disease progression and determining the optimal cut-off values.* AUCs of SUV_max_ (a), MTV (b), and TLG (c) were 0.48, 0.76, and 0.67, respectively. The optimal cut-off values for PFS derived from the AUC data were MTV 45 ml (sensitivity: 78%, specificity: 75%) and TLG 150 SUV*∗*ml (sensitivity: 78%, specificity: 60%).

**Figure 2:**
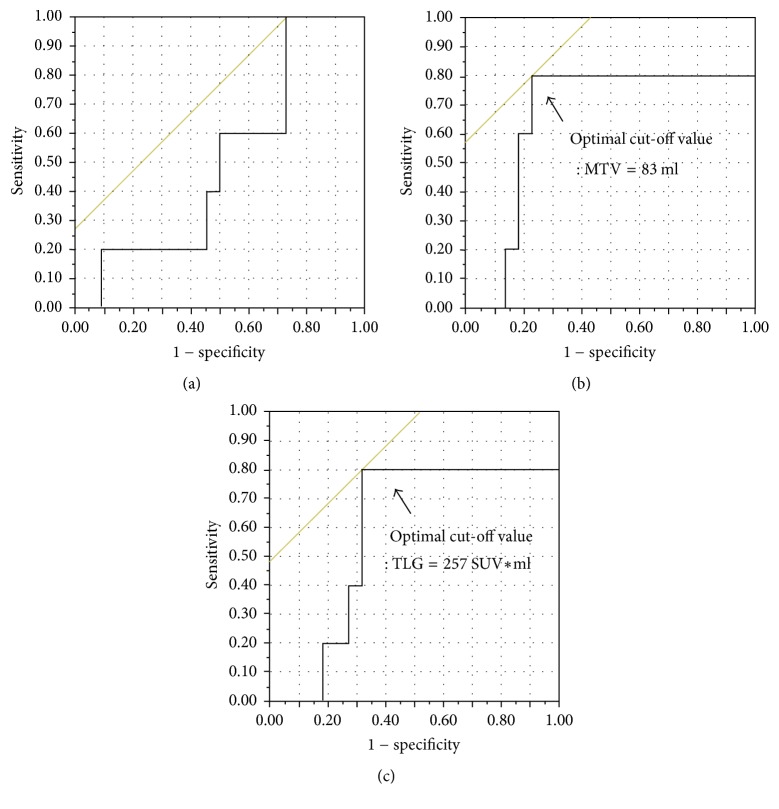
*ROC curve analysis comparing the prognostic accuracy for overall survival and determining the optimal cut-off values.* AUCs of SUV_max_ (a), MTV (b), and TLG (c) were 0.50, 0.65, and 0.58, respectively. The optimal cut-off values for PFS derived from the AUC data were MTV 83 ml (sensitivity: 80%, specificity: 73%) and TLG 257 SUV*∗*ml (sensitivity: 80%, specificity: 68%).

The mean SUV_max_ of the primary lesions was 8.4 ± 6.2 SUV (median = 6.11) and the mean MTV and TLG of the primary lesions were 56.6 ± 59 ml (median = 40.2) and 250 ± 269 SUV*∗*ml (median = 150), respectively. The abilities of the SUV_max_, MTV, and TLG values for various SUV thresholds to predict PFS were calculated by their ROC curves (Figure 1). The area under the curve (AUC) of SUV_max_ was 0.48, suggesting that SUV_max_ would be inappropriate to evaluate the clinical outcome of PMSTs. On the other hand, the AUC values of MTV and TLG were 0.76 and 0.67, respectively. The optimal cut-off values for PFS derived from the AUC data were MTV 45 ml (sensitivity: 78%, specificity: 75%) and TLG 150 SUV*∗*ml (sensitivity: 78%, specificity: 60%). Meanwhile, the abilities of the SUV_max_, MTV, and TLG values to predict OS were also calculated by their ROC curves and we found that the AUCs of SUV_max_, MTV, and TLG were 0.50, 0.65, and 0.58, respectively. The optimal cut-off values for OS derived from the AUC data were MTV 83 ml (sensitivity: 80%, specificity: 73%) and TLG 257 SUV*∗*ml (sensitivity: 80%, specificity: 68%) (Figure 2).

### 3.3. Kaplan-Meier Survival Estimates

**Figure 3:**
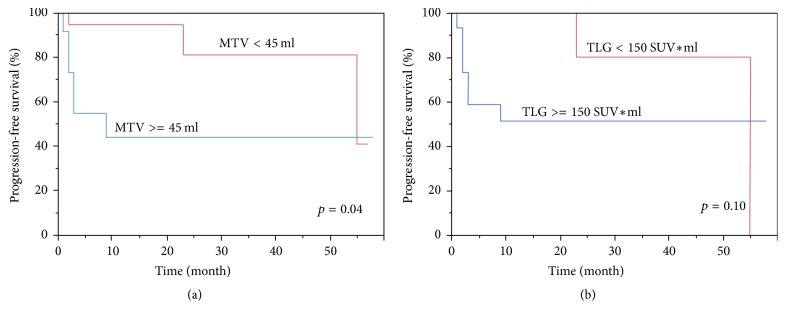
*Kaplan-Meier estimate of progression-free survival by MTV and by TLG.* Data were categorized according to the optimal cut-off value for MTV (a) and TLG (b) defined with ROC curve analysis.

Patients were divided according to the below and above cut-off value for MTV and TLG. We found that MTV were identified as significant prognostic factor for PFS by log-lank test (*p* = 0.04). In addition, TLG was not significantly correlated with PFS (*p* = 0.10) (Figure 3).

**Figure 4:**
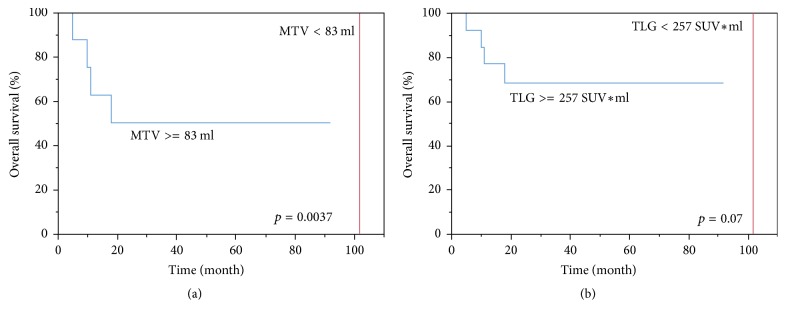
*Kaplan-Meier estimate of overall survival by MTV and by TLG.* Data were categorized according to the optimal cut-off value for MTV (a) and TLG (b) defined with ROC curve analysis.

We also observed that MTV, but not TLG, was significantly correlated with OS (*p* = 0.0037 and 0.07, resp.) (Figure 4).

### 3.4. Prognostic Values of the Metabolic Parameters

The univariate analysis with variables affecting PFS demonstrated that MTV above the optimal discriminating cut-off value was associated with poor outcome (*p* = 0.04). In the multivariate analysis, MTV above the optimal discriminating cut-off value was the only significant prognostic factor for PFS (HR 14.6 [95% CI 1.78–333]), *p* = 0.01 (Table 2). In addition, the univariate analysis with variables affecting OS demonstrated that MTV and TLG above the optimal discriminating cut-off value were associated with poor outcome (*p* = 0.002 and 0.03, resp.). In the multivariate analysis, MTV, but not TLG, above the optimal discriminating cut-off value was significantly associated with poorer OS (HR 46.1 [95% CI 1.20–216]), *p* = 0.035 (Table 3).

### 3.5. Case Presentation

**Figure 5:**
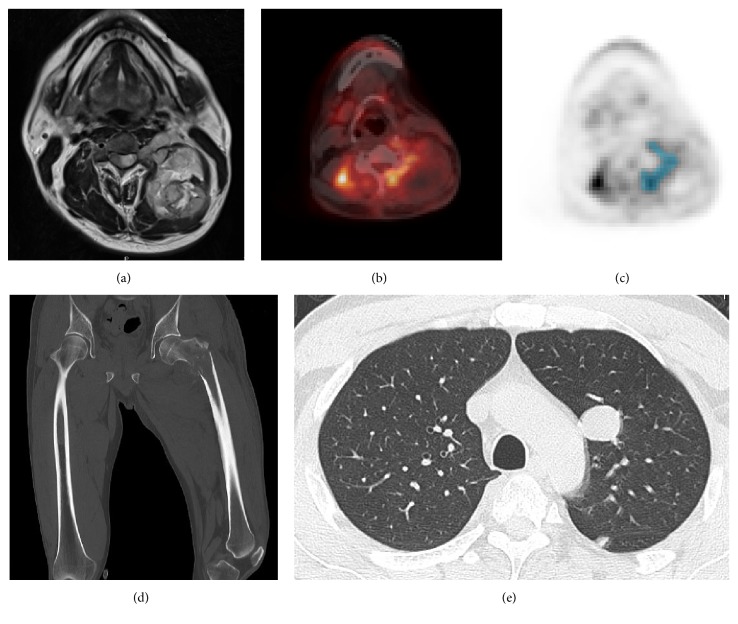
*Representative case presentation of SUV*_*max*_ *and metabolic parameters in large and heterogeneous cervical spinal tumor.* A spinal MPNST in a 40-year-old man. (a) The mass was 9 cm in size and the axial T2-weighted MRI showed mixed intense signal mass with unclear boundary in epidural and paravertebral space. (b) A preoperative ^18^F-FDG PET/CT scan was obtained and the tumor showed moderate SUV_max_ (5.25 g/mL). (c) For calculations of metabolic parameters, a volume-of-interest was drawn on the PET images (light blue area). A preset threshold of 2.5 of SUV of the tumor was used to define the MTV (84.3 mL) and the mean SUV of the MTV was determined (SUV_mean_ 3.06 SUV). MTV and SUV_mean_ were used to calculate the TLG (258 SUV*∗*ml). The patient underwent a partial resection of the epidural tumor and carbon-ion radiotherapy. Five months after surgery, multiple bone (d) and lung metastasis (e) were detected and the patient died 9 months after surgery.

An example of relative discordance between SUV_max_ and metabolic parameters is a spinal MPNST in the cervical spine in a 40-year-old man. The mass was 9 cm in size and the axial T2-weighted MRI showed mixed intense signal mass with unclear boundary in epidural and paravertebral space (Figure 5(a)). A preoperative ^18^F-FDG PET/CT scan was obtained and the tumor showed moderate SUV_max_ (5.25 g/mL) (Figure 5(b)). For calculations of metabolic parameters, a volume-of-interest was drawn on the PET images (light blue area) (Figure 5(c)). A preset threshold of 2.5 of SUV of the tumor was used to define the MTV (84.3 ml) and the mean SUV of the MTV was determined (SUV_mean_ 3.06 SUV). MTV and SUV_mean_ were used to calculate the TLG (258 SUV*∗*ml). The patient underwent a partial resection of the epidural tumor by posterior approach. Subsequently, he was treated by carbon-ion radiotherapy. Five months after surgery, multiple bone (Figure 5(d)) and lung (Figure 5(e)) metastasis were detected and the patient died 9 months after surgery.

## 4. Discussion

One of the commonly used systems to predict the prognosis of the malignant tumors is AJCC stage system. This system is based on anatomical and histologic information and proved to be a simple and reliable predictor of tumor outcomes [16]. However, the AJCC stage system is not suitable to evaluate the prognosis of PMSTs [17]. Importantly, the introduction of FDG PET has offered the possibility of noninvasive estimation of biological activity of malignant tumors and it also may help the predication of patient outcome. Conventionally, SUV_max_ has been applied widely to predict the prognosis and treatment outcomes [6, 7].

However, SUV_max_ reflects only the most active part of the tumor and it does not represent the overall characteristics of the tumor, particularly the tumor with heterogeneous features [14]. For instance, sarcomas commonly present with mixed high- and low-grade areas since they contained various mesenchymal elements including myxoid substance, osteoid, chondroid matrix, and necrosis [18], suggesting that SUV_max_ would be suboptimal to assess the biological activity of sarcomas, including PMSTs. Consistent with this, a study of 238 sarcoma patients showed the lower predictive value of SUV_max_ compared to the new algorithm for considering the heterogeneous ^18^F-FDG spatial distribution in sarcoma [7].

The volume-based ^18^F-FDG imaging markers, MTV and TLG, have theoretical advantage in terms of evaluating the total volume and activity of metabolically active tumor cells compared to SUV_max_. This has been confirmed by several studies showing significant prognostic properties of MTV and TLG for prediction of clinical outcome in the patients with various malignant tumors [10, 19]. However, reported data regarding the application of MTV and TLG for sarcoma patients are conflicting. One study reports the superiority of TLG to MTV as a significant predictor of progression-free survival in soft-tissue sarcomas [14]. On the contrary, Byun et al. [20] failed to demonstrate the superiority of TLG to MTV as an independent prognostic value in patients with osteosarcomas of the extremities. Remarkably, MTV with a fixed SUV threshold of 2.0, but not TLG, is identified as a predictive factor for metastasis-free survival in that cohort. Our results also postulated that, for predicting progression of PMSTs, MTV is more accurate than TLG.

The plausible explanation for the discrepancy between the results of the above-mentioned studies is that the location of the included tumors is different between the studies. We focused on the spine/spinal tumors and curative surgeries were achieved only in 5 cases (19%). On the contrary, the previous study included tumors located in the extremities that can basically be resected with wide margin [14]. Therefore, in our cases, the residual tumor burden after initial treatment would be bigger than the tumors in extremities and MTV might reflect more accurately the “real tumor burden” compared to TLG.

The present study has several limitations. First, the study was a retrospective design and enrolled only a small number of subjects by the low incidence of PMSTs. Second, the method for measuring and calculating MTV needs standardization and refinement. For example, differences in SUV measurements in different PET scanners may preclude the application of MTV in routine and reproducible clinical practice. In addition, although we set 2.5 of SUV as the margin threshold for calculating MTV in this study, it might not be the optimal threshold. Third, we applied ROC curve analysis to find the optimized cut-off for prediction of prognosis, PFS. However, this method can easily induce overcorrected results and we should be careful in interpreting the results. Together, prospective studies in a larger population are warranted to validate MTV as the robust predictive factor for clinical outcomes of patients with PMSTs.

In conclusion, MTV may be a more accurate predictor of PFS and OS in PMSTs compared to TLG or SUV_max_. We anticipate that MTV offers pretreatment assessment of disease activity of PMSTs and helps decision-making for guiding rational treatment options. The predictive efficacy of MTV in diverse clinical settings, such as evaluation of treatment response, should be validated in the future studies.

## Figures and Tables

**Table 1:** Clinical characteristics of patients.

Characteristics	Value
Total number of patients	27 (100%)
Sex	
Male	13 (48%)
Female	14 (52%)
Age (years) mean (range)	53.9 (12–82)
Location	
Cervical	7 (26%)
Thoracic	10 (37%)
Lumbar	6 (22%)
Sacral	4 (15%)
Size	
≧5 cm	14 (52%)
<5 cm	13 (48%)
Histology	
MPNST	5
UPS	4
Osteosarcoma	3
Chondrosarcoma	3
Leiomyosarcoma	2
Chordoma	2
GCTB	2
Others	6
Surgery	
Total	16 (59%)
with wide margin	5
Chemotherapy	15 (56%)
Radiotherapy	16 (59%)

MPNST: malignant peripheral nerve sheath tumor, UPS: undifferentiated pleomorphic sarcoma, and GCTB: giant cell tumor of bone.

**Table 2:** Factors affecting progression-free survival in the univariate and multivariate analyses.

Factor	Cut-off value	Univariate	Multivariate		
		*p* value	HR	95% CI	*p* value
MTV	45 ml	0.04	14.6	1.78–333	0.01
TLG	150 SUV*∗*ml	0.12			
Size	5 cm	0.05	2.97	0.69–11.8	0.14
Surgery	Yes	0.78			
Surgery with wide margin	Yes	0.42			
Chemotherapy	Yes	0.03^#^	1.67	0.50–5.69	0.40
Radiotherapy	Yes	0.36			

HR: hazard risk. 95% CI: 95% confidence interval. ^#^Progression-free survival was negatively associated with the administration of chemotherapy (*p* = 0.03), which may indicate the aggressiveness of the tumors of the patients who had chemotherapy.

**Table 3:** Factors affecting overall survival in the univariate and multivariate analyses.

Factor	Cut-off value	Univariate	Multivariate		
		*p* value	HR	95% CI	*p* value
MTV	83 ml	0.002	46.1	1.20–216	0.035
TLG	257 SUV*∗*ml	0.03	1	0.99–1.02	0.99
Size	5 cm	0.35			
Surgery	Yes	0.95			
Surgery with wide margin	Yes	0.25			
Chemotherapy	Yes	0.59			
Radiotherapy	Yes	0.65			

HR: hazard risk. 95% CI: 95% confidence interval.
